# Combined herbal fumigation and fire acupuncture for refractory plantar warts: A retrospective study

**DOI:** 10.12669/pjms.42.2.13807

**Published:** 2026-02

**Authors:** Fengming Qi, Dan Deng, Liang Tian

**Affiliations:** 1 Fengming Qi Department of Dermatology, Hangzhou Linping District Integrated, Traditional Chinese and Western Medicine Hospital, Hangzhou, Zhejiang Province 310002, P.R. China; 2 Dan Deng Department of Dermatology, Hangzhou Linping District Integrated, Traditional Chinese and Western Medicine Hospital, Hangzhou, Zhejiang Province 310002, P.R. China; 3 Liang Tian Department of Rehabilitation Medicine, Zhejiang Provincial People’s Hospital, Hangzhou, Zhejiang Province 310014, P.R. China

**Keywords:** Chinese Herbal Fumigation Steam Therapy, Fire acupuncture, Plantar warts, Refractory, Traditional Chinese medicine

## Abstract

**Objective:**

To explore the efficacy and safety of Chinese Herbal Fumigation Steam Therapy (CHFST) combined with fire acupuncture in the treatment of refractory plantar warts.

**Methodology:**

This retrospective cohort study was conducted at the Hangzhou Linping District Integrated Traditional Chinese and Western Medicine Hospital. A total of 126 patients with refractory plantar warts who received treatment from March 2022 to March 2025 were included. Of them, 63 patients who received CHFST combined with fire acupuncture (observation group) were matched with the cohort receiving cryotherapy (control group) in a 1:1 ratio. The primary outcome was the clinical efficacy assessed three months after treatment. The secondary outcome was the improvement in immune indicators and the occurrence of adverse reactions.

**Results:**

After three-month follow-up, the clinical efficacy of the observation group was significantly higher than that of the control group (*P*<0.05). In both groups, the post-treatment levels of T lymphocytes (CD8^+^, CD3^+^, CD4^+^ and CD4+/CD8+) were higher than those before treatment. Post-treatment levels of CD3^+^, CD4^+^ and CD4^+^/CD8^+^ in the observation group were significantly higher than those in the control group (*P*<0.05). There was no statistically significant difference in the incidence of adverse reactions between the two groups (*P*>0.05).

**Conclusions:**

The study confirms that CHFST, when combined with fire acupuncture, is more effective than conventional cryotherapy for treating refractory plantar warts.

## INTRODUCTION

Plantar warts are benign hyperkeratotic lesions of the soles caused by human papillomavirus (HPV) infection.^1^,^2^ Contributing factors include trauma, friction, and excessive foot sweating.^1^ Plantar warts that resist treatment after several months of conventional therapy, referred to as refractory plantar warts, mainly manifest as the proliferation of keratin in the plantar area, with loose and soft cores visible after keratin removal, often accompanied by small black dots due to capillary bleeding.^2^ Standard treatment methods for refractory warts include surgical destruction, chemoimmunotherapy, laser therapy, electrocautery and liquid nitrogen cryotherapy.^3^-^7^

However, the efficacy of these methods is limited. Cryotherapy remains one of the most commonly used treatments and has a well-established therapeutic effect.^4^,^5^ However, as an invasive therapy, cryotherapy is tissue-destructive, associated with slow wound healing and may cause adverse reactions such as secondary infection and pigment changes, along with a high risk of recurrence.^4^,^5^ Treating plantar warts with the CO_2_ laser, which acts on tissues non-selectively and has strong penetrability, is often associated with damage to adjacent tissues and scar formation.^6^ Surgical excision fails to address the issue of subclinical infection with human papillomavirus (HPV), leading to a high risk of recurrence after surgery.^7^

In recent years, Traditional Chinese Medicine (TCM) treatment methods for plantar warts have become exceedingly popular.^8^-^10^ Fire acupuncture, a commonly used TCM treatment in dermatology, has been demonstrated as an effective treatment for plantar warts.^8^ However, fire acupuncture alone has a relatively long treatment course and its efficacy is poor especially for thickened and refractory lesions on the feet.^9^ According to TCM theory, plantar warts are caused by qi and blood stasis and invasion of wind-heat toxin pathogens taking advantage of deficiency. Thus, treatments should focus on clearing heat and detoxifying, reducing swelling and relieving pain, etc.^8^,^9^ Wang et al.^10^ used Chinese Herbal Fumigation Steam Therapy (CHFST) to treat plantar warts using the prescription containing ingredients such as Portulaca oleracea L., Cyrtomium fortunei J. Sm. and Isatis indigotica Fortune, which are beneficial for effectively soothing the liver and regulating qi, harmonizing qi and blood, etc. Additionally, these ingredients have certain antiviral effects, which is conducive to improving the efficacy of plantar wart treatment. However, when CHFST is used alone for refractory plantar warts, the thick stratum corneum on the feet makes it difficult for drugs to penetrate into the deep layer of the lesion, resulting in an excessively long treatment course that some patients find hard to adhere to.^11^

At present, the efficacy of CHFST combined with fire acupuncture in the treatment of some dermatological diseases has been confirmed.^12^,^13^ However, there are no reports on the use of CHFST combined with fire acupuncture for the treatment of refractory plantar warts. This study aimed to analyze the efficacy and safety of CHFST combined with fire acupuncture in the treatment of refractory plantar warts.

## METHODOLOGY

This retrospective cohort study was conducted at Hangzhou Linping District Integrated Traditional Chinese and Western Medicine Hospital and included 126 patients with refractory plantar warts treated between March 2022 and March 2025. Sixty-three patients who underwent Chinese Herbal Fumigation Steam Therapy (CHFST) combined with fire acupuncture were matched in a 1:1 ratio with 63 patients who received cryotherapy. Frequency matching was applied based on age (±5 years), gender, body mass index (±2 kg/m^2^), disease duration (±3 months), number of warts (±1), and lesion diameter (±1 mm) to reduce baseline imbalance. All patients met the diagnostic criteria for refractory plantar warts and fulfilled predefined inclusion and exclusion criteria. Patients were retrospectively identified from electronic medical records. Treatment allocation was not influenced by patient or physician preference but was determined by the availability and timing of therapies—cryotherapy being more commonly used in the earlier period of the study, while CHFST combined with fire acupuncture was introduced later following institutional approval. Although prior treatment type (such as cryotherapy, topical salicylic acid, laser ablation, or immunotherapy) was not used as a matching variable, chart review indicated that their distribution was generally comparable between the two groups.

### Ethical approval:

The study was approved by the Ethics Review Committee of Hangzhou Linping District Integrated Traditional Chinese and Western Medicine Hospital (2022-Z-007) on December 29, 2022. Due to the retrospective nature of the study, informed consent was waived.

### Inclusion Criteria:

- Aged 16 to 60 years.
- Meeting the diagnostic criteria for plantar warts.^2^
- Having a diagnosis of refractory plantar warts, defined as warts that persisted for six months or more, failed to respond to at least one full course of standard treatment, and had a minimum clearance period of three months following the last intervention.^2^,^6^
- No local redness, ulceration, or erosion of the skin lesions.
- No cognitive impairment and completion of a three-month follow-up.

### Exclusion criteria:

- Patients taking glucocorticosteroids, immunosuppressants or antibiotics for a long time.
- Patients with autoimmune diseases, severe heart, liver, kidney or hematopoietic system diseases, or diabetes.
- Patients with scar constitution.
- Pregnant and lactating women.

### Treatment Methods:

### Cryotherapy:

- A disposable surgical blade was used to precisely trim and remove the hyperplastic tissue of the wart and the thickened stratum corneum at the edge of the wart along the skin plane. Trimming was performed until the skin had a moderate hardness and softness, was smooth without edges or corners and no bleeding occurred.
- After trimming, liquid nitrogen was used for cryotherapy on the wart, with the appearance of a pale circle at the edge as the endpoint response. Trimming and cryotherapy were performed once every two weeks.

### CHFST Combined with fire acupuncture:

- Trimming was performed as described above.
- After trimming, the lesion was disinfected. An acupuncture needle (0.30 × 40mm) was heated until red-hot over an alcohol lamp. The lesion was accurately located, and the needle was inserted vertically into its base, with 3-5 consecutive insertions until the top of the wart formed a scab. A 1cm area around the edge of the wart was subjected to shallow needling (0.25 - 0.5mm), with a needling interval of 0.2 cm. No foot bathing was allowed on the day of treatment. Trimming and fire acupuncture were performed once every two weeks.

Starting from the second day, CHFST was administered daily. The prescription included: Galla Chinensis 30g, Portulaca oleracea L. 30g, Equisetum hiemale L. 30g, vinegar-processed Cyperus rotundus L. 30g, Patrinia scabiosaefolia Fisch. ex Trevir. 30g, Angelica sinensis (Oliv.) Diels 20g, Prunus persica (L.) Batsch 30g and Coptis chinensis Franch. 15g. After decocting the TCM herbs, the lesion was first fumigated with the steam. When the temperature dropped to 42°C, the herb residues were not removed and the decoction was poured into a constant-temperature foot bath for external washing. CHFST was performed once a day, with a total duration of 30 minutes each time. All fire acupuncture procedures were performed by the same experienced dermatologist, who holds a certified license in Traditional Chinese Medicine and has over 10 years of clinical experience in fire needle therapy, particularly in dermatological conditions. To ensure consistency and reduce inter-operator variability, a standardized operating procedure (SOP) was strictly followed throughout the treatment. This protocol included predefined parameters such as needle heating time, insertion depth (2–3 mm for the core of the lesion and 0.5 mm around the periphery), insertion spacing (0.2 cm), and post-procedural care. The operator also underwent procedural training and calibration prior to study initiation to enhance technical uniformity and minimize bias related to practitioner performance.

### Follow-up:

Treatment was administered continuously for three months. After the end of the treatment course, follow-up was conducted for another three months, with one follow-up visit per month.

### Primary outcomes:

Clinical responses were evaluated by two dermatologists using a blind method at each visit. Clinical digital photographs of the lesions were taken at baseline, at each subsequent visit and at the final follow-up. Referring to the Criteria for Diagnosis and Therapeutic Effect of TCM Diseases and Syndromes,^12^ therapeutic effects were classified as follows: Cure: complete regression of warts; Marked effect: regression of warts > 70%; Effective: regression of warts between 30% and 69%; Ineffective: regression of warts < 30% or even enlargement. The total effective rate was calculated as follows: Total effective rate = (Number of cases with cure + marked effect + effective) / Total number of cases × 100%.

### Secondary Outcomes:

Improvement of T lymphocyte subset levels: A 4ml fasting venous blood sample was collected from the patient. Serum levels of CD8^+^, CD3^+^, CD4^+^ and CD4^+^/CD8^+^ ratio were detected using a BD FACSCalibur flow cytometer. Adverse reactions included mild burning sensation, infection, redness and swelling, ulceration and superficial scars.

### Statistical analysis:

Statistical analysis was performed using SPSS version 24.0 (IBM Corp., Armonk, NY, USA). Descriptive statistics were calculated for all variables. The Kolmogorov–Smirnov test, combined with visual inspection of histograms and Q–Q plots, was used to assess the normality of continuous variables. CD3^+^ and CD4^+^ levels (before and after treatment) were approximately normally distributed, while variables such as CD4^+^/CD8^+^ ratio and disease duration exhibited non-normal distribution. For normally distributed or approximately normal variables, independent-samples t-tests were used for between-group comparisons. For non-normally distributed variables, the Mann–Whitney U test was used, and the Wilcoxon signed-rank test was applied for within-group paired comparisons where appropriate. Categorical variables were analyzed using the chi-square (χ^2^) test. The primary analysis compared the total effective rate between the two groups using the chi-square test. A significance threshold of P < 0.05 was considered statistically significant. Test statistics (t, Z, or χ^2^) and exact P-values are reported where applicable. Additionally, a post hoc power analysis was conducted based on the observed difference in total efficacy (92.1% vs. 79.4%). Using a two-sided chi-square test with α = 0.05 and a total sample size of 126 (63 per group), the estimated statistical power was 81.3%, indicating that the study was adequately powered to detect the observed difference.

## RESULTS

The study included clinical records of 126 patients (56 males and 70 females). The age range of the cohort was 17-60 years, with an average age of 42.4 ± 11.1 years. Sixty-three patients who received CHFST combined with fire acupuncture were matched with the cohort receiving cryotherapy in a 1:1 ratio. There was no statistically significant difference in baseline data between the two patient groups (P > 0.05) (Table-I).

**Table-I:** Comparison of baseline data between two groups.

Baseline data	Observation group (n=63)	Control group (n=63)	χ^2^/t/U/Z	SMD	P
Gender			0.289	0.13	0.591
Male	30 (47.6)	26 (41.3)			
Woman	33 (52.4)	37 (58.7)			
Age (years)	40.8±11.7	44.0±10.3	-1.595	-0.28	0.113
BMI (kg/m^2^)	23.4±3.2	22.9±2.8	0.872	0.16	0.385
Disease duration (months)	15 (9-25)	13 (9-19)	1.507	0.27	0.134
Number of plantar warts	4 (2-5)	3 (2-5)	0.824	0.15	0.412
Lesion size (mm)	6 (4-7)	5 (5-6)	0.718	0.13	0.474

***Note:*** Data are presented as mean ± SD for normally distributed continuous variables and median (IQR) for non-normally distributed variables. Independent-samples t-test was used for approximately normal variables, Mann–Whitney U test for non-normal variables, and chi-square test for categorical variables. SMD = standardized mean difference.

After three-month follow-up, the observation group reported 21 cases of cure, 29 cases of marked effect and eight cases of effective, with a total effective rate of 92.1%. In the control group, 13 cases were cured, 28 cases showed significant improvement and nine cases were effective, with a total effective rate of 79.4%. The total effective rate in the observation group was significantly higher than that in the control group (P < 0.05) (Table-II).

**Table-II:** Comparison of clinical efficacy rates between two groups.

Group	Cure	Marked effect	Effective	Ineffective	Total effective rate
Observation group (n=63)	21 (33.3)	29 (46.1)	8 (12.7)	5 (7.9)	58 (92.1)
Control group (n=63)	13 (20.6)	28 (44.5)	9 (14.3)	13 (20.6)	50 (79.4)
*χ^2^*					4.148
*P*					0.042

***Note:*** Values are expressed as number (percentage). Chi-square test was used to compare categorical distributions between groups. Total effective rate = Cure + Marked effect + Effective.

Before treatment, there was no significant difference in the levels of CD8^+^, CD3^+^, CD4^+^ and CD4^+^/CD8^+^ between the two groups (*P*>0.05). Post-treatment levels of CD8^+^, CD3^+^, CD4^+^ and CD4^+^/CD8^+^ in both groups were significantly higher than before treatment. The levels of CD3^+^, CD4^+^ and CD4^+^/CD8^+^ in the observation group were significantly higher than those in the control group (*P*<0.05) (Table-III).

**Table-III:** Comparison the levels of CD8^+^, CD3^+^, CD4^+^ and CD4^+^/CD8^+^ between two groups.

Indexes	Observation group (n=63)	Control group (n=63)	χ^2^/t/Z	P
***Before treatment***				
CD8^+^	24.7±5.4	23.4±5.7	1.353	0.179
CD3^+^	61.3±5.2	59.9±6.1	1.389	0.167
CD4^+^	36.3±3.9	34.7±5.2	1.943	0.054
CD4^+^/CD8^+^	1.55 (1.26-1.73)	1.50 (1.27-1.87)	1930	0.792
***After treatment***				
CD8^+^	25.6±5.1	24.4±5.6	1.243	0.216
CD3^+^	69.5±6.0	64.4±5.1	5.226	<0.001
CD4^+^	45.3±4.6	38.4±5.5	7.559	<0.001
CD4^+^/CD8^+^	1.82 (1.54-2.12)	1.61 (1.41-1.84)	2495	0.013

***Note:*** Data are presented as mean ± SD or median (IQR) depending on distribution. Independent-samples t-test was used for approximately normally distributed variables (CD3^+^, CD4^+^), while Mann–Whitney U test was used for non-normally distributed variables (CD4^+^/CD8^+^ ratio). P-values are based on between-group comparisons.

During the treatment period, the observation group experienced three cases of mild burning sensation, two cases of infection, one case of redness and swelling, two cases of ulcers and one case of superficial scar, with a total incidence rate of 14.3% (9/63). The control group experienced one case of slight burning sensation, two cases of infection, one case of redness and swelling and one case of ulcer, with a total incidence rate of 7.9% (5/63). There was no statistically significant difference in the incidence of adverse reactions between the two groups (*P*>0.05) (Table-IV). All adverse events were classified as mild in severity. No moderate or severe events occurred in either group. None of the adverse reactions led to treatment discontinuation or required systemic medical intervention. Minor events such as ulceration or burning sensation were managed with saline cleansing and topical anti-inflammatory ointments. No patient required hospitalization or exhibited long-term sequelae.

**Table-IV:** Comparison of adverse reaction rates between Two Groups.

Adverse event	Observation group (n=63)	Control group (n=63)	χ^2^	P
Slight burning sensation	3 (4.8)	1 (1.6)		
Infection	2 (3.2)	2 (3.2)		
Redness and swelling	1 (1.6)	1 (1.6)		
Ulcer	2 (3.2)	1 (1.6)		
Superficial scar	1 (1.6)	0 (0)		
Total number of occurrences	9 (14.3)	5 (7.9)	1.286	0.257

***Note:*** Data are expressed as number of events (percentage of patients). Chi-square test was used for group comparisons of adverse event rates. All adverse events were graded as mild in severity. No patient discontinued treatment or required hospitalization. Minor events were managed with simple topical care.

## DISCUSSION

This retrospective study demonstrates that Chinese Herbal Fumigation Steam Therapy (CHFST) combined with fire acupuncture is a safe and more effective treatment for refractory plantar warts compared to cryotherapy. The observation group showed significantly higher clinical efficacy, more pronounced improvement in immune parameters (CD3+, CD4+, CD4+/CD8+), and no increase in adverse event rates compared to the control group. To our knowledge, few studies have systematically evaluated the combination of CHFST and fire acupuncture for the treatment of refractory plantar warts.

Our findings are consistent with prior studies on fire acupuncture and CHFST. Zhang et al.^12^ applied fire needle and CHFST for psoriasis and reported a 96% effectiveness rate at six-month follow-up. He et al.^13^ observed improved skin lesion repair and sleep quality in elderly patients with pruritus using the same combination. The dual properties of fire acupuncture, acting as both acupuncture and moxibustion, may explain these outcomes. The mechanism includes warming meridians, unblocking collaterals, promoting qi and blood circulation, and detoxifying.^14^ The thermal stimulation damages the wart tissue and its vascular base, disrupting nutrient supply, which leads to regression.^14^–^16^ CHFST further supports treatment by softening the stratum corneum, enhancing drug penetration, and promoting absorption.^17^,^18^

From the TCM perspective, plantar warts result from wind-heat invasion and qi-blood stagnation. Therefore, clearing heat and removing toxins is central to treatment. In modern immunological terms, “heat-clearing” corresponds to anti-inflammatory effects and cytokine suppression; “detoxification” corresponds to pathogen elimination via antigen presentation and cytotoxic T lymphocyte activation. Key herbs in the CHFST formulation, such as Portulaca oleracea L. and Coptis chinensis Franch., exhibit proven antiviral and immunomodulatory effects.^18^ These mechanisms may underlie the observed increase in CD3+, CD4+, and CD4+/CD8+ ratios in the treatment group.

The immune system plays a critical role in the progression and treatment resistance of plantar warts. In immunocompromised patients, HPV replicates more readily and tends to recur after treatment.^1^–^3^ HPV colonization of keratinocytes and glandular ducts also complicates eradication.^2^–^4^ Tao et al.^19^ reported that local immune function is associated with spontaneous wart regression. Thus, enhancing local immunity is essential in treating refractory plantar warts.

In this study, both groups experienced improved T-cell subsets after treatment, but the CHFST + fire acupuncture group exhibited significantly greater enhancement. This suggests superior immunomodulatory effects of the combination therapy. The fire needle’s thermal action may destroy necrotic wart cells and release viral antigens, prompting immune activation.^20^,^21^ CHFST complements this by promoting circulation, dispersing nodules, and enhancing skin immune defense.^12^,^13^,^22^ Other pharmacologic effects include increased tissue metabolism, lymphatic flow, accelerated necrosis and apoptosis of wart cells, suppression of pathogen growth, and facilitation of tissue repair.^23^,^24^

This treatment regimen may be particularly suitable for patients with recurrent or refractory plantar warts who do not respond well to conventional cryotherapy or are intolerant to its side effects. Because CHFST and fire acupuncture are relatively low-cost and can be administered in TCM-capable community or outpatient clinics, this combined therapy shows promise for broader clinical implementation, especially in resource-limited or integrative medicine settings.

### Strength of study:

It include its direct comparison of two clinical treatment regimens, incorporation of immune biomarkers, and real-world patient data. However, limitations include its retrospective design, limited follow-up duration, and absence of blinding, which may introduce bias. Furthermore, correlation between immune changes and clinical outcomes could not be analyzed due to data stratification limitations, and is recommended for future prospective studies.

In terms of safety, there was no statistically significant difference in adverse reaction incidence between groups. All events were mild and included slight burning, infection, redness, ulcer, or superficial scar. Transient burning is a common response to thermal or cryogenic stimulation and typically resolves within hours. Minor infections likely stem from impaired skin barrier and can be prevented by topical antisepsis. Redness and swelling may arise from deeper stimulation or hypersensitivity and can be treated with cold compresses or anti-inflammatory ointments. Ulcers may occur due to excessive penetration or improper post-treatment care.

In terms of tolerability, patients undergoing fire acupuncture occasionally reported transient discomfort or burning sensations, primarily during or shortly after the procedure. For those with low pain thresholds, topical lidocaine gel was applied before treatment. Patients were instructed to avoid intense physical activity on the day of therapy. Importantly, no cases of functional limitation (such as impaired walking or work absenteeism) were reported, suggesting that the therapy is well tolerated in daily life.

### Limitations:

As a retrospective, non-randomized analysis with a moderate sample size, it may be subject to selection bias and unmeasured confounders such as lesion severity, previous treatment types, pain threshold, and patient expectations. Despite baseline matching, these variables could have influenced treatment response. Future randomized, controlled studies with stratification are necessary to verify the results. The short follow-up period—limited to three months—precludes assessment of long-term efficacy and recurrence, particularly given the known relapsing nature of plantar warts. Extended prospective observation (six to twelve months) is needed to clarify the durability of treatment effects. Patient-centered outcomes such as pain intensity, functional recovery, and quality of life were not evaluated. These measures are critical in assessing acceptability, especially for interventions involving physical discomfort like fire acupuncture. Their absence reflects the constraints of retrospective data. Incorporating standardized instruments such as pain scales and functional assessments will strengthen future evaluations. The CHFST protocol administered daily for 30 minutes over three months poses practical challenges. While clinical observations suggest good adherence, documentation was inconsistent. Prospective studies should evaluate compliance rates and explore more flexible regimens to improve feasibility in routine care. Accurate localization of plantar wart lesions can be difficult due to their irregular borders, requiring dense and skilled needling for adequate treatment. Operator experience thus plays a role in treatment success, underscoring the importance of technical standardization. Although most patients tolerated fire acupuncture well, transient pain or discomfort was occasionally reported. Further investigation into pain mitigation strategies is warranted to improve patient comfort and adherence. Finally, the study did not examine the relationship between changes in immune cell subsets and clinical outcomes. This precludes mechanistic interpretation of immune improvements. Prospective studies incorporating immune monitoring alongside clinical response categorization will help elucidate this connection and refine therapeutic strategies.

## CONCLUSION

This study shows that the combination of CHFST with fire acupuncture is an effective method to treat refractory plantar warts. This treatment plan is safe, cost-effective and can be easily promoted in medical institutions at all levels. This combined therapy may be particularly suitable for patients with refractory plantar warts who are intolerant to cryotherapy or have experienced frequent recurrences. Given its low cost and reliance on available Traditional Chinese Medicine resources, it is especially feasible for implementation in community-level or integrated medicine clinics, making it a promising option for wider clinical use.

### Authors’ contributions:

**FQ:** Literature search, study design and manuscript writing.

**DD & LT:** Data collection, data analysis and interpretation. Critical Review.

**FQ:** Manuscript revision and validation and is responsible for the integrity of the study.

All authors have read and approved the final version of manuscript.
